# Recent advances in understanding and managing psoriatic arthritis

**DOI:** 10.12688/f1000research.9592.1

**Published:** 2016-11-16

**Authors:** Dafna D. Gladman

**Affiliations:** 1 Psoriatic Arthritis Program, Centre for Prognosis Studies in the Rheumatic Diseases, Toronto Western Hospital, Toronto, Ontario, M5T 2S8, Canada

**Keywords:** psoriasis, PsA, TNF inhibitors, CASPAR

## Abstract

This article reviews recent advances in psoriatic arthritis (PsA) over the past several years with emphasis on early diagnosis, better understanding of pathogenesis, and new therapeutic approaches. Early diagnosis is important, since people who present late do not fare as well. There are a number of clinical, laboratory, and ultrasound features that can help identify patients destined to develop PsA, and several screening tools have been developed. It is recognized that genetic and epigenetic factors, as well as T cells and cytokines, play a role in the pathogenesis of PsA, and several targets have been identified for therapeutic interventions. New therapies have been developed and tested in PsA and have been found to be highly effective for both skin and joint manifestations of the disease.

The expectation is that, in the future, PsA patients will be treated early and more aggressively and that there will not be significant progression of joint damage. Moreover, with effective treatment of the skin and joint disease and management of risk factors for the comorbidities, we can expect to reduce their occurrence and further reduce the excess mortality and reduced quality of life and function in these patients.

## Introduction

Psoriatic arthritis (PsA) is an inflammatory musculoskeletal disease associated with psoriasis ^1^. Psoriasis is an inflammatory skin condition manifesting with scaly erythematous skin lesions occurring usually on the extensor surfaces of the elbows and knees but may affect the scalp, inter-gluteal area, umbilical area, and other parts of the body ^2^. Psoriasis occurs in 2–3% of the population, and about 30% of patients with psoriasis develop PsA. PsA affects men and women equally, and the average age at onset is in the patient’s fourth decade. PsA involves the peripheral joints (arthritis), axial skeleton (spondylitis), insertion of tendons and ligaments into bone (enthesitis), inflammation of whole digits (dactylitis), skin, and nails ^3^.

In its initial descriptions, PsA was considered a mild form of arthritis. However, over the past few decades, it has become clear that the disease is more common and more severe than previously thought. PsA may result in joint damage leading to marked disability and reduced quality of life. It is associated with increased mortality risk ^4^. Progression of joint damage and mortality are related to inflammatory burden as well as previous damage. The diagnosis of PsA was difficult because of its heterogeneity, but the development of the classification criteria for PsA (Classification of PsA [CASPAR]) has facilitated the diagnosis ^5^. Several screening tools have also been developed to increase recognition of the disease among dermatologists and primary care physicians.

Until 2000, the treatment for PsA was not very effective and, despite the recognition of the prognostic factors, there was not much that could be done. However, with the advent of biologic therapy, there are now potent treatments available for patients with PsA. Moreover, recent studies have demonstrated that early diagnosis is important if we are to prevent damage in patients with PsA. Additional therapeutic agents have become available such that the life of the patient with PsA can now be modified.

This article reviews recent advances in PsA over the past several years with emphasis on early diagnosis, better understanding of pathogenesis, and new therapeutic approaches.

## Importance of early diagnosis

### Why do we need to diagnose psoriatic arthritis early?

As noted above, PsA is associated with progressive joint damage, reduced quality of life and function, and increased mortality ^4^. These untoward outcomes are related to preceding joint inflammation and accrued damage. It stands to reason that if we treat patients aggressively with the appropriate medications to control inflammation and prevent damage in the first place, we would improve their quality of life and function and allow a normal life span. Indeed, two recent studies support this notion. A study performed at the University of Toronto PsA clinic demonstrated that patients who were reviewed in the clinic within 2 years of PsA diagnosis fared better than those who were first evaluated in the clinic with longer disease duration ^6^. The longer the disease duration prior to the patient’s first visit to the PsA clinic, the higher the risk of developing joint damage. A study performed in the PsA clinic in Dublin, Ireland, provided even more compelling evidence for the need to diagnose patients with PsA early. Haroon *et al*. demonstrated that even a 6-month delay in rheumatologic consultation (from the onset of symptoms) resulted in worse outcome for patients with PsA, with higher health assessment questionnaire (HAQ) scores, more erosions, and sacroiliitis ^7^. Thus, it seems that the earlier that PsA patients are diagnosed, the better the outcome.

We have also recognized recently that patients with PsA suffer from a number of comorbidities, many of which are precipitated by persistent inflammation. Patients with PsA have an increased frequency of cardiovascular disease, diabetes, the metabolic syndrome, and depression ^8^.

### How do we diagnose psoriatic arthritis early?

***Clinical features.*** There are several clinical features that may identify patients with psoriasis destined to develop arthritis ^9– 12^. The extent of psoriasis is higher among patients diagnosed with PsA compared to psoriasis patients without arthritis (psoriasis cutaneous [PsC]). The location of psoriasis, especially involvement of the scalp and inter-gluteal areas, has also been reported to occur more commonly among patients with PsA than uncomplicated psoriasis. However, most dermatologists believe that scalp and inter-gluteal lesions are so common in psoriasis that they would not help identify those patients who should be referred to a rheumatologist. On the other hand, nail lesions occur in over 80% of patients with PsA compared to only about 40% of PsC patients.

In a prospective study of 464 patients with psoriasis who were confirmed not to have inflammatory arthritis at presentation to the clinic, 51 developed PsA during an 8-year follow-up, for an annual incidence of 2.7%. Baseline variables identified as risk factors for the development of PsA included severe psoriasis, low level of education, and the use of retinoids. Using a time-dependent analysis, nail pitting and uveitis remained significant in a multivariate model ^12^.

***CASPAR criteria.*** The CASPAR criteria should also help to identify PsA early. While the criteria were established in patients who had long-standing disease, they work just as well in patients with early disease ^13– 16^. However, the CASPAR criteria are based on the stem of inflammatory musculoskeletal disease. Only rheumatologists can accurately make that diagnosis. To address this issue, the Group for Research and Assessment of Psoriasis and PsA (GRAPPA) is developing criteria to identify inflammatory arthritis that can be used by non-experts ^17^. Since it is not feasible for all patients with psoriasis to be reviewed by a rheumatologist, several groups have developed screening tools that can be administered to patients.

### Screening tools for psoriatic arthritis

A number of screening tools were developed specifically for patients with psoriasis to identify those who have PsA ^18– 20^. Two tools were developed for screening for PsA in the general population ^21^. However, although all of these screening tools were very sensitive and specific in their development programs, when screening tools were compared in independent settings from those in which they were developed, they did not function very well ^22^.

***Ultrasound.*** The use of ultrasound may be helpful in identifying patients with PsA early, particularly among patients with psoriasis. Gisondi *et al*. performed an ultrasound study of entheses in 30 patients with psoriasis and 30 controls ^23^. They found that the entheses were thicker and the overall ultrasound score was higher in patients with psoriasis than in controls. They repeated the ultrasound assessment among the psoriasis patients 2 years later, and three of the 30 had developed PsA ^24^. However, a study that compared ultrasound in patients with PsA, patients with PsC, and healthy controls found that obesity is a confounder in distinguishing between the groups ^25^.

***Biomarkers.*** Since psoriasis usually precedes the development of PsA, and dermatologists have difficulty identifying inflammatory arthritis, it would be helpful if clinicians had a biomarker that would identify those individuals likely to develop the disease. In the past few years, we have seen several biomarkers tested for PsA. These include genetic, epigenetic, soluble, and cellular biomarkers ^26, 27^.

Among the genetic biomarkers, human leukocyte antigen (HLA) alleles that distinguish patients with PsA from those with PsC have been identified and replicated. In a study of 712 patients with PsA and 335 patients with PsC, Eder *et al*. ^28^ demonstrated that the HLA alleles B*08, B*27, and B*38 are risk factors for the development of PsA, whereas HLA-C*06 is “protective”. Similar observations were reported from the Dublin cohort, where it was also reported that the presence of HLA-B*27 was associated with the early development of PsA among patients with psoriasis but the presence of HLA-C*06 was associated with a delayed onset of PsA ^29^. Data from the immunochip studies in psoriatic disease confirmed differences between psoriasis and PsA at the HLA region and suggested that HLA-B amino acid position 45 may be driving this heterogeneity ^30^. Major histocompatibility complex (MHC) class I chain-related A (MICA) alleles have been associated with PsA; however, a study comparing PsA patients to PsC patients demonstrated that only homozygosity for the allele MICA*00801 was associated with PsA ^31^.

Recent genome-wide association studies (GWAS) have identified single-nucleotide polymorphisms (SNPs) near HLA-C, tumor necrosis factor (TNF) receptor superfamily member 9 (TNFRSF9), and late cornified envelope 3A (LCE3A) as being more strongly associated with psoriasis than PsA, whereas SNPs near interleukin 23 receptor (IL-23R) and TNF alpha-induced protein 3 (TNFAIP3) were more strongly associated with PsA than with PsC ^32^. Protein tyrosine phosphatase, non-receptor type 22 (PTPN22) was also found to distinguish patients with PsA from those with psoriasis alone ^33^. Gene expression profiling of patients with PsA and PsC also identified potential genes as biomarkers for PsA, including Notch 2 N-Terminal Like ( *NOTCH2NL*), histone acetyltransferase 1 ( *HAT1*), C-X-C motif chemokine ligand 10 ( *CXCL10*), and SET domain containing 2 ( *SETD2*) ^34^.

Soluble biomarkers may be more accessible in a clinic setting. Chandran *et al*. ^35^ found that levels of osteoprotegerin, high-sensitivity C-reactive protein (hs-CRP), cartilage oligomeric matrix protein (COMP), matrix metalloproteinase 3 (MMP-3), and the ratio of C-propeptide of type II collagen (CPII) to collagen fragment neoepitopes Col2-3/4 (C2C ratio) were higher in patients with PsA versus PsC. Two of these biomarkers, MMP-3 and hs-CRP, were replicated in a study that compared PsA patients to healthy controls, which also identified vascular endothelial growth factor as a potential screening tool for the detection of PsA ^36^. More recently, *CXCL10*, which has been identified in a microarray analysis as being a gene associated with PsA, was identified as a soluble biomarker for developing PsA among psoriasis patients followed prospectively ^37^. Psoriasis patients who subsequently developed PsA had higher levels of CXCL10 in the serum than those who did not develop PsA. Moreover, CXCL10 levels decreased in the serum after the development of PsA. A comparison of paired serum and synovial fluid (SF) samples from patients with PsA demonstrated higher levels in the SF compared to blood, suggesting that *CXCL10* may be a biomarker for the development of PsA in psoriasis patients as well as having a pathogenetic role in the development of the disease.

Proteomic analyses of SF have also been performed to identify candidate biomarkers for PsA. Cretu *et al*. identified 137 proteins that were differentially expressed between PsA and control SF, of which 44 were upregulated ^38^. The expression of 12 proteins (myeloperoxidase [MPO], Mac-2-binding protein [M2BP], defensin alpha 1 [DEFA1], histone 4 [H4], histone 2A type I A [H2AFX], orosomucoid 1 [ORM1], CD5-like protein [CD5L], profilin 1 [PFN1], C4b-binding protein [C4BP], MMP-3, S100 calcium-binding protein A9 [S100A9], and CRP) was subsequently confirmed to be elevated in PsA SF using selected reaction monitoring assays. These markers are being investigated in the serum as potential biomarkers for PsA ^39^.

Cellular biomarkers have also been identified. An increased frequency of osteoclast precursors (OCPs) was found in one-third of patients with PsC and in the majority of patients with PsA ^40^. An increase in OCP correlated with erosive disease. The same group developed an antibody against a dendritic cell-specific transmembrane protein (DC-STAMP), which correlated with OCPs and may be another biomarker to identify patients with PsA early ^41^. These biomarkers are currently being investigated in patients with psoriasis who progress to develop PsA.

Thus, a number of biomarkers are being sought that can identify patients with psoriasis who are destined to develop PsA. It is most likely that there will not be one biomarker but rather a combination of biomarkers that can be applied to most accurately identify patients with PsA early.

## What have we learnt about the pathogenesis of psoriatic arthritis?

Although the exact pathogenesis of PsA is not known, genetic, immunologic, and environmental factors are thought to play a role ^42, 43^.

### Genetic factors

Among genetic factors, the HLA loci have been the most persistently documented. Two recent case control studies confirmed the association between PsA and HLA-B*27, HLA-B*38, HLA-B*39, and HLA-C*06 compared to healthy controls ^29, 44^. HLA-C*0602 is the allele most strongly associated with psoriasis and is also a risk factor for PsA. While HLA-C*0602 is associated with an earlier diagnosis of psoriasis, it is associated with a later diagnosis of PsA ^29^.

Non-HLA genes have also been implicated, both within and outside the MHC. Killer immunoglobulin receptor (KIR) genes, particularly *KIR2DS2*, have also been proposed as susceptibility genes for PsA ^45^. These genes are coded on chromosome 19 but use the HLA-C molecules as ligands.

GWAS have identified 40 loci in patients with psoriasis, many of which are related to immune function, including the endoplasmic reticulum aminopeptidase 1 (ERAP1), whose product is relevant to peptides binding to the MHC class I molecules, especially HLA-C*0602 and HLA-B*27 ^46^. SNPs related to genes relevant to immune function include loci containing genes involved in nuclear factor kappa-light-chain-enhancer of activated B cells (NF-kB) signaling ( *REL*, TNF α-induced protein 3-interacting protein 1 [ *TNIP1*], NF-kB inhibitor alpha [ *NFKBIA*], and caspase recruitment domain family member 14 [ *CARD14*]), interferon (IFN) signaling (interleukin 28 receptor A [ *IL-28RA*] and tyrosine kinase 2 [ *TYK2*]), T-cell regulation (Runt-related transcription factor 3 [ *RUNX3*], interleukin 13 [ *IL-13*], T-cell activation RhoGTPase activating protein [ *TAGAP*], ever shorter telomeres protein 1 [ *ETS1*], and methyl-CpG binding domain protein 2 [ *MBD2*]), antiviral signaling (IFN-induced helicase C1 protein [‎ *IFIH1*], DEXD/H-box helicase 58 [ *DDX58*], and ring finger protein 114 [ *RNF114*]), and genes involved in the IL-23 pathway which specifically implicate a role for Th17 cells ( *TNFAIP3*, *IL-23R*, *IL-12B*, TNF receptor associated factor 3 interacting protein 2 [ *TRAF3IP2*], *IL-23A*, and signal transducer and activator of transcription 3 [ *STAT3*]). Most of these have also been identified in PsA, but only two of these loci were independently identified in PsA, namely IL-12B and IL-23R, with the IL-23R SNP being independent to the SNP found in psoriasis alone, and another region on chromosome 5q31 has also been identified as a marker for PsA ^32, 47^.

Many of the genes listed above encode proteins that are important in the immune response, suggesting that genetic factors may exert their effect through immune mechanisms.

### Immunological factors

The association with class I HLA alleles, the presence of activated CD8+ T cells and natural killer (NK) cells in the psoriatic synovium, and the response of the disease to immunomodulatory therapy indicate that the immune system, especially the lymphocytes, plays an important role in PsA pathogenesis ^48^. Both the innate and the adaptive immune responses are implicated in the pathogenesis of PsA. KIR receptors are found on NK cells and use the HLA molecules as a ligand, implicating the innate immune system. Increased levels of cytokines have been detected in the peripheral blood, skin, and synovium of patients with PsA. While the role of T cells has been suspected for many years, more recently the role of Th17 cells and the IL-17 axis has been elucidated.

### Environmental factors

The association between environmental factors and psoriatic disease has long been known. Streptococcus infection followed by pustular psoriasis has been described. More recently, two studies confirm the association between infection and the development of PsA. Pattisson *et al*. compared the prevalence of environmental exposures among 98 British PsA and 163 psoriasis patients over a window of exposure that ranged from 5–10 years prior to the onset of arthritis ^49^. They identified physical trauma, rubella vaccination, oral ulcers, and moving house were found to be associated with PsA. Subsequently, another case control study was performed by Eder *et al*. in which they administered structured questionnaires to 159 patients with recent-onset PsA and 159 patients with PsC and found that infections that required antibiotic treatment, injuries, and occupations that involved lifting heavy weights were associated with PsA, while there was an inverse association with smoking ^50^. A population-based study identified obesity as a risk for developing PsA ^51^.

## What are the advances in therapies for psoriatic arthritis?

Conventional disease-modifying anti-rheumatic drugs (DMARDs) do not work well in PsA. A number of systematic reviews have been carried out and demonstrate that the effect size of methotrexate, sulfasalazine, and leflunomide are not very high and cyclosporine is toxic ^52^. Fortunately for PsA patients, TNF inhibitors (TNFi) became available at the beginning of the millennium and have demonstrated excellent efficacy in patients with PsA ^53^. Five TNFi agents are now available, including etanercept, adalimumab, infliximab, golimumab, and certolizumab ^54^. Certolizumab is the most recent addition to the list and has proven to be efficacious in TNFi failures ^55^. All have been proven to be effective for signs and symptoms as well as for preventing radiographic progression of PsA. Infliximab, golimumab, and certolizumab have been proven to be effective for enthesitis and dactylitis in pivotal randomized controlled trials (RCT), while etanercept and adalimumab have been shown to control enthesitis and dactylitis in subsequent studies ^54^.

More recently, the anti-IL-12/-23 antibody ustekinumab was proven to be effective for PsA. While its efficacy for the arthritis is not quite as high as that of the anti-TNF agents, it works very well for the psoriasis. It does work for dactylitis and enthesitis as well ^56^. Next came the phosphodiesterase-4 (PDE4) inhibitor apremilast. Four phase III trials were carried out, demonstrating efficacy against placebo in patients with a history of conventional DMARD failure ^57^. Apremilast proved efficacious for peripheral arthritis, although not quite as effective as the anti-TNF agents. It was also effective for dactylitis and enthesitis. However, its effect on radiographic progression was not tested in these trials.

Based on the role of the Th17 axis in PsA, antibodies to the IL-17A cytokine were developed. Secukinumab was tested in two phase III placebo-controlled RCTs. The first, FUTURE-1, used 10 mg/kg secukinumab intravenously at weeks 0, 2, and 4, followed by subcutaneous secukinumab at a dose of either 150 mg or 75 mg every 4 weeks, or placebo ^58^. Primary outcome was American College of Rheumatology 20% (ACR20) response at 24 weeks. Significantly higher responses were observed for the two drug-treated groups compared to placebo. Secondary end points, including the American College of Rheumatology 50% (ACR50) response and joint structural damage, were significantly better in the secukinumab groups than in the placebo group. FUTURE-2 included three doses: 75, 150, and 300 mg. While there were loading doses, in this study they were subcutaneous, not intravenous ^59^. The 75 mg dose did not work as well as the higher doses for the joints. The 300 mg dose was clearly better for the skin. Importantly, in both trials, secukinumab was effective for both TNFi-naïve and TNFi-experienced patients, although the 300 mg dose was more effective for the latter.

A recent systematic review summarizes therapies for PsA and provides the level of evidence for the efficacy of each of the drugs, together with their level of efficacy ^54^. Results of the SPIRIT-1 study, which tested ixekizumab, another anti-IL-17A agent, in a phase III RCT which included adalimumab as an active comparator, were recently published ^60^. In this study, patients with active PsA were randomized to ixekizumab 80 mg every 2 weeks, ixekizumab every 4 weeks, adalimumab 40 mg every 2 weeks, or placebo. The study demonstrated the efficacy of ixekizumab in controlling arthritis, skin and nail disease, dactylitis, and enthesitis as well as structural damage. This drug is now awaiting approval.

### Therapies under investigation

Several studies are currently ongoing in PsA. Abatacept, or CTLA-Ig, a selective T-cell costimulation modulator that is approved for the treatment of rheumatoid arthritis, was initially tested in a phase II study which demonstrated efficacy as well as reduction in MRI inflammation. A phase III study has just been completed and the results should be available soon. The Janus Kinase (JAK) inhibitor tofacitinib, also available for the management of rheumatoid arthritis, has also been tested in PsA. The results of these studies will be available soon. An IL-23 antibody has also been tested in psoriasis and will be tested in PsA.

## Summary and future directions

Recent advances in PsA have emphasized that the disease is more common and more severe than previously thought. We now know that even a 6-month delay in consultation with a rheumatologist results in untoward effects. Since PsA usually occurs after the development of psoriasis, we must screen patients with psoriasis for the development of PsA so that they can be identified and treated early. A number of screening tools have been developed, which include clinical features, screening questionnaires, and biomarkers, but an algorithm needs to be developed to accurately identify these patients early.

New information on the pathogenesis of PsA has led to the development of new therapeutic interventions. We now have several anti-TNF agents, anti-IL-12/-23 agents, and anti-IL-17 agents. Additional agents are currently under investigation and will hopefully be approved soon.

Therefore, we can expect that patients with PsA will be treated early and more aggressively and that there will not be significant progression of joint damage. Moreover, with effective treatment of the skin and joint disease and management of risk factors for the comorbidities, we can expect to reduce their occurrence and further reduce the excess mortality and reduced quality of life and function in these patients.

## Abbreviations

CASPAR, Classification of Psoriatic Arthritis; CXCL10, C-X-C motif chemokine ligand 10; DMARD, disease-modifying anti-rheumatic drug; GRAPPA, Group for Research and Assessment of Psoriasis and Psoriatic Arthritis; GWAS, genome-wide association studies; HAQ, health assessment questionnaire; HLA, human leukocyte antigen; hs-CRP, high-sensitivity C-reactive protein; IFN, interferon; IL-12B, interleukin 12 beta; IL-23R, interleukin 23 receptor; KIR, killer immunoglobulin receptor; MHC, major histocompatibility complex; MICA, major histocompatibility complex class I chain-related A; MMP-3, matrix metalloproteinase 3; NF-kB, nuclear factor kappa-light-chain-enhancer of activated B cells; NK, natural killer; OCP, osteoclast precursor; PsA, psoriatic arthritis; PsC, psoriasis cutaneous (cutaneous psoriasis without arthritis); RCT, randomized controlled trial; SF, synovial fluid; SNP, single-nucleotide polymorphism; TNF, tumor necrosis factor; TNFAIP3, tumor necrosis factor alpha-induced protein 3; TNFi, tumor necrosis factor inhibitor.
